# Hypofractionated Proton Boost Combined with External Beam Radiotherapy for Treatment of Localized Prostate Cancer

**DOI:** 10.1155/2012/654861

**Published:** 2012-07-08

**Authors:** Silvia Johansson, Lennart Åström, Fredrik Sandin, Ulf Isacsson, Anders Montelius, Ingela Turesson

**Affiliations:** ^1^Section of Oncology, Department of Radiology, Oncology and Radiation Science, Uppsala University Hospital, 751 85 Uppsala, Sweden; ^2^Regional Cancer Centre, Uppsala Orebro, 751 85 Uppsala, Sweden; ^3^Section of Medical Physics, Department of Radiology, Oncology and Radiation Science, Uppsala University Hospital, 751 85 Uppsala, Sweden

## Abstract

Proton boost of 20 Gy in daily 5 Gy fractions followed by external beam radiotherapy (EBRT) of 50 Gy in daily 2 Gy fractions were given to 278 patients with prostate cancer with T1b to T4N0M0 disease. Fifty-three percent of the patients received neoadjuvant androgen deprivation therapy (N-ADT). The medium followup was 57 months. The 5-year PSA progression-free survival was 100%, 95%, and 74% for low-, intermediate-, and high-risk patients, respectively. The toxicity evaluation was supported by a patient-reported questionnaire before every consultant visit. Cumulative probability and actuarial prevalence of genitourinary (GU) and gastrointestinal (GI) toxicities are presented according to the RTOG classification. N-ADT did not influence curability. Mild pretreatment GU-symptoms were found to be a strong predictive factor for GU-toxicity attributable to treatment. The actuarial prevalence declined over 3 to 5 years for both GU and GI toxicities, indicating slow resolution of epithelial damage to the genitourinary and gastrointestinal tract. Bladder toxicities rather than gastrointestinal toxicities seem to be dose limiting. More than 5-year followup is necessary to reveal any sign of true progressive late side effects of the given treatment. Hypofractionated proton-boost combined with EBRT is associated with excellent curability of localized PC and acceptable frequencies of treatment toxicity.

## 1. Introduction

One of the first-line curative treatment options of prostate carcinoma is radiotherapy (RT), which can be considered for organ-confined disease as well as for locally advanced tumors. The message of the potential benefit of dose escalation for disease-free survival announced by Hanks [1] and Perez et al. [2] from retrospective studies became a milestone for the current successful outcome in the use of radiotherapy for curative intent. Since then, several mature randomized trials have proven that dose escalation up to 79 Gy to the prostate is advantageous with regards to clinical disease control [3–14]. Further dose escalation to 81 Gy delivered in a phase II study using intensity modulated radiotherapy (IMRT) resulted in excellent 8-to-10-year tumors control and acceptable late toxicity [15, 16]. A feasibility study of proton beam therapy (PBT) suggested that daily proton fractions of 2 Gy to 82 Gy might be the maximal dose that can be delivered with acceptable late morbidity [17]. It is noteworthy that in phase II studies, to 81 and 82 Gy the dose was prescribed to the planning target volume (PTV). That means a 5 to 10% higher dose if prescribed according to isocenter, which was the case in the majority of the dose escalation trials up to 79 Gy.

The high fractionation sensitivity proposed for prostate cancer implies that the use of fewer large dose fractions might be an alternative to dose escalation using daily fractions of 2 Gy. Hypofractionation is attractive because of sparing of patient-treatment visits and due to cost-effectiveness. Most interestingly, in the case that fraction-size sensitivity is significantly higher for prostate cancer than for nearby dose-limiting normal tissues, larger dose fractions will increase tumor control probability without increasing toxicity. Since the first proposal of a low *α*/*β* value for prostate cancer presented by Brenner and Hall, 1999, [18], a lot of clinical research effort was put into this issue. In 2008, the status about the *α*/*β* values and available clinical information on efficacy and toxicity in the use of hypofractionation was critically reviewed by Miles and Robert Lee. They also presented an overview of ongoing randomized trials [19]. To our knowledge, only one prospectively designed randomized trial based upon an *α*/*β* of 1.5 Gy and isoeffective total doses to 80 Gy in 2 Gy fractions is published. So far hypofractionation with 3.1 Gy was superior to conventional fractionation in terms of biochemical control for high-risk prostate cancer, without increasing toxicity [20].

In this paper we report the 5-year outcome of a hypofractionation regimen based on a proton boost with dose fractions of 5 Gy. The physical characteristics of the proton beams for therapy (particle therapy) differ from the photon beams. Proton beam therapy will characteristically give a peak (the Bragg peak) in the dose distribution at a depth close to the maximum range of the protons. Beyond the maximum range, the dose drops rapidly to zero. Proton beams have reduced lateral scatter compared to photons at depths below 10 cm. At larger depths the side scatters increase resulting in penumbras of the same magnitude as for high-energy photons. The characteristics of the proton beam can be used to give a high dose to the tumor target and at the same time keep the dose to surrounding tissues low. Proton beam therapy therefore offers an opportunity of dose escalation to the prostate without increasing the dose delivery to normal tissues. Generally, proton beam dose distributions can be made more conformal to the tumor target than photon beam dose distributions delivered by various IMRT techniques [21, 22].

The proton facility at The Svedberg Laboratory in Uppsala, Sweden, was, for the first time in the world, utilized by a group led by Stig Sténson and Börje Larsson to treat the patients with cancer [23, 24]. The longest experience of using proton beam therapy to treat prostate cancer has Massachusetts General Hospital and Loma Linda University Medical Centre [3, 8, 25, 26].

With the attractive physical features of proton beams and the possible advantage of large dose fractions in prostate cancer, a treatment schedule with a hypofractionated PBT boost added to conventional radiotherapy was introduced by the equipment at hand for physical research at The Svedberg Laboratory in Uppsala. The intention was, for the sake of logistical and medical reasons, to establish an alternative to dose escalation with high dose-rate brachytherapy (HDR-BT), mainly in use for patients with prostate carcinoma in our department. The Regional Ethical Review Board in Uppsala approved the analysis of treatment outcome.

## 2. Materials and Methods

Between November 2002 and late December 2008, 278 patients with histologically confirmed prostate adenocarcinoma received proton boost of 20 Gy in daily 5 Gy fractions followed after one-week rest by external beam radiotherapy (EBRT) of 50 Gy in daily 2 Gy fractions. Assuming a value of *α*/*β* of 3 Gy or 1.5 Gy and a value of relative biological effectiveness (RBE) of 1.1, the equivalent dose in 2 Gy fractions (EQD2) for the schedule would be 87 Gy or 94 Gy, respectively, to the prostate. Including the RBE correction the proton dose per fraction is 5.5 GyE. Thirteen patients were lost in followup. The characteristics of 265 patients are listed in Table 1. The median age at diagnosis was 65 years (range, 46–77 years).

The T-classification was based on digital rectal examination. Bone scintigraphy was used to exclude bone metastases. Surgical obturatory lymph node dissection or magnetic resonance imaging (MRI) was used for pelvic lymph node staging. Risk group classification was performed according to the NCCN guidelines [27]. Patients were classified in three risk categories according to T-stage, Gleason score, and the prostate-specific antigen (PSA) level at diagnosis. Low risk was defined as clinical stage of T1b-T1c-T2a tumor, PSA < 10 ng/mL, and Gleason score ≤ 6. Intermediate risk was classified as having T2b-T2c tumor, Gleason score of ≤7, and PSA < 20 ng/mL. Patients were classified as high risk if they had tumors of T3a or a higher stage, Gleason score ≥ 8, or PSA > 20 ng/mL. The majority of the patients had high (40%) or intermediate risk (36%). Only 63 patients (24%) were classified as low risk.

Bladder, bowel, and sexual function assessments by the physician and a patient self-administered questionnaires were performed before the start of any therapy and at any follow-up visit. The questionnaire translated from SOMA-LENT covering scoring (score 0 to 10) of functions and symptoms from the genitourinary and gastrointestinal tract, including sexual function [28]. The information in these questionnaires was for the present analysis converted to the RTOG grading system for genitourinary-(GU-) and gastrointestinal-(GI-) pretreatment symptoms and treatment induced side effects [29]. At baseline, that is, (before treatment start), grade 0, 1, 2, and 3 for GU-symptoms was reported in 122 (46.5%), 95 (35.8%), 38 (14.3%), and 9 (3.4%) patients, respectively. Grades 0, 1, and 2 GI pretreatment symptoms were reported in 244 (92.4%), 15 (5.7%), and 5 (1.9%), respectively, (Table 1).

The median volume of the prostate gland measured by transrectal ultrasound at diagnosis was 37 cc (range, 14–120 cc) for the whole patient cohort. Patients with low- intermediate-, or high-risk profile had a median prostate volume of 31 cc (range, 19–90 cc), 40 cc (range, 14–113 cc), and 37.5 cc (range, 14–120 cc), respectively. The number of patients with prostate volumes ≥50 cc was 72 of which 43 had larger than 60 cc.

The median PSA for patients with low-, intermediate-, or high-risk profile was 6.1 ng/mL (range, 3–9.6 ng/mL), 10.5 ng/mL (range, 1.7–19.9 ng/mL), and 18 ng/mL (range, 3.6−158 ng/mL), respectively. Eight patients had PSA > 50 ng/mL.

Neoadjuvant androgen deprivation therapy (N-ADT) was given to 139 out of 265 patients (53%). Ninety-nine patients out of 139 received four weeks of bicalutamide treatment followed by gonadotropin-releasing hormone (GNRH) after two weeks. Forty out of 139 patients had only received bicalutamide treatment (150 mg) due to request to preserve sexual function. N-ADT was prescribed for 22% of the low-risk and 45% of the intermediate-risk patients for a median time of 5 months. Eighty-one (76%) patients with high-risk profiles received N-ADT to a median time of 7.6 months.

Patients were followedup at intervals of 3 to 4 months for 2 years, than 6 months up to 5 years, and thereafter yearly. The median followup was 57 months (range, 5.8–109 months) calculated from the start of EBRT. Biochemical failure (PSA-relapse) was defined according to the RTOG-ASTRO Phoenix criteria as the absolute nadir PSA level plus 2 ng/mL [30]. Persisting toxicity was defined as any symptom occurring >6 months after the start of proton treatment.

### 2.1. Radiotherapy

For guidance of the delineation of the clinical target volume (CTV), all patients underwent a diagnostic 1.5 Tesla magnetic resonance imaging (MRI). Three or four radiopaque markers had been inserted in the prostate under local anesthesia using a transperineal applicator before the treatment planning computerized tomography (CTs) were performed. The proton boost and the EBRT were delivered in different treatment positions, in lithotomy and supine position, respectively. For the treatment with proton boost, patients were immobilized with an in-house constructed couch for transperineal treatment with a horizontal-fixed proton beam. For the latest 147 patients of the whole cohort a rectal retraction rod was fixed into a length of 7-8 cm of the rectum. The rectal retraction rod was used both at the CT imaging and during each proton treatment. Positioning and fixation with the rectal retraction rod, containing 3 radiopaque markers, and its impact on the dose distribution of the patients were described in more detail elsewhere [31].

All patients were CT-scanned in both treatment positions from L5-S1 levels to approximately 10 cm caudal to the ischial tuberosities on the same day. CT-slice thickness was 2 mm for proton dose planning and 3–5 mm for photon dose planning. The image data for proton planning were introduced into the Helax-TMS (Treatment Management System, Nucletron, Uppsala, Sweden) and for dose planning of the EBRT into the Oncentra Treatment Planning System (Nucletron, Uppsala, Sweden). The bladder, the rectum, and the penile bulb were considered as critical normal tissues.

The definitions of target volumes were based on the publications from the International Commission on Radiological Units and Measurements (ICRU) Reports 62 and 78. The CTV included only the prostate for the proton boost to low- and intermediate-risk patients. The prostate and the proximal part (1.5 cm) of the seminal vesicles were delineated as the CTV for the high-risk group [32]. The planning target volume (PTV) was defined when a margin of 3 mm was added for low-risk patients, and 5 mm for intermediate- and high-risk patients to the CTV in all directions, except to the area between the prostate and rectum. Here 2 mm was used for all three risk categories. Concerning EBRT, the CTV was defined as the prostate for the low-risk group. The prostate including the whole seminal vesicles defined the CTV for the intermediate- and high-risk groups. The PTV for the EBRT was defined as the CTV plus a margin of 15 mm in lateral and ventral directions and 10 mm in cranial, caudal, and dorsal directions for all risk groups.

For the planning of the proton boost the range compensation filters were calculated to obtain a distal dose distribution conformal to the PTV. For this purpose an extra 10 mm beam margin was added in longitudinal direction. This allows for range uncertainties in the variable entrance region (±5 mm), in bolus construction (±2 mm), and accelerator energy (±1 mm).

On four consecutive days (Tuesday to Friday), a dose of 20 Gy in 5 Gy fractions was administrated as a boost to the prostate with protons at The Svedberg Laboratory in Uppsala as a single, fixed, horizontal beam with an energy of 180 MeV. In the perineal beam entrance an individually shaped aperture was applied. The radiopaque markers allow verification of the position both of the prostate and the rectal rod by CT-imaging for treatment planning. They were also used for positioning of the daily proton fraction by orthogonal X-ray imaging [31].

One week after the completion of the proton boost treatment, the EBRT was started with daily 2 Gy fractions (Monday to Friday) for 5 weeks, in total 50 Gy. The EBRT was delivered with an Elekta Precise linear accelerator (Elekta AB, UK) equipped with a multileaf collimator (MLC), using 15 megavolt (MV) X-rays. A three-field technique with 2 opposed lateral fields and an anterior field, all equally weighed, was used. Thirteen patients (12%) of the high-risk group were treated with intensity-modulated radiotherapy (IMRT) to the prostate including the seminal vesicles and pelvic lymph nodes with daily 2 Gy fractions up to 50 Gy during a 5-week period.

Portal images for EBRT were used to verify the position by matching to bony structures. The gold markers could not be used to verify the position as the small marker size used for proton therapy made them hard to visualize on MV portal imaging. The less accurate positioning of the prostate for EBRT compared to proton therapy motivated the larger PTV margin for photon beam treatment.

The dose limitation to the anterior rectum wall was set to 70 Gy, in terms of EDQ2 with *α*/*β* of 3 Gy, to a volume less than 10 cc. The reference for the anterior rectal wall was to the point halfway between the inner and outer rectum contour on the CT. The dose maximum volume was always located just behind the prostate.

### 2.2. Design of the Proton Boost Treatment and Patient Selection

The background to the design of the proton boost dose escalation was that patients with a large prostate volume, above 55 to 60 cc, or width of 5.5 cm, or larger are not suitable for the HDR-BT technique. This is the case because the intended needle positions could not be reached due to the conflict with bone structures. Patients not eligible for a boost with the HDR-BT technique were treated with a conformal 3-field photon technique with daily 2 Gy fractions to 70 Gy to the prostate with a wide margin. Additionally, 2 fractions of 2 Gy were added as a boost to the prostate with a narrow margin, delivering a total dose to the prostate of 74 Gy. These were the treatment options for radiotherapy of prostate cancer up to November 2002 at the Department of Oncology, Uppsala University Hospital.

The HDR-BT boost technique with 2 fractions of 10 Gy combined with EBRT of 50 Gy given in daily 2 Gy fractions results in a physical dose of totally 70 Gy. However, the biological effectiveness of this schedule in terms of EDQ2, when using an *α*/*β* value of 3 Gy or 1.5 Gy is 102 Gy or 117 Gy (minimum dose), respectively, with inhomogeneous dose distribution. Compared to this dose the conventional photon technique with 74 Gy might have a dramatically lower efficacy if the *α*/*β* value is truly low. In the light of the paper by Pollacket al. published 2002 [4], showing evidence for the benefit of dose escalation from 70 to 78 Gy in a phase III randomized study, we were challenged to find a better treatment option for the patient cohort not eligible for the HDR-BT boost.

 We approached the problem by considering the use of our physical proton facility, available only 1 week per month for patient treatments. Mondays were reserved for preparation and simulation procedures; therefore, we started with proton boost of 4 fractions. The proton boost was followed by one-week gap to keep the overall treatment time to 7 weeks, in order to be equal to the HDR-BT boost schedule. Moreover, we wanted to keep the physical dose with the proton and HDR-BT boost equal, that is, the proton boost was given with 4 daily fractions of 5 Gy and total dose for both techniques was 70 Gy. The corresponding EDQ2 with *α*/*β* of 3 Gy or 1.5 Gy and RBE 1.1 was 87 Gy or 94 Gy, respectively, and with a homogeneous dose distribution. Thus, the proton boost technique resulted in a dose escalation, far more satisfactory than the dose escalation obtained with our conventional photon treatment of 74 Gy, but still lower than for our standard HDR-BT boost technique.

After the 10 first pilot proton boost treatments, the majority of patients who were also eligible for HDR-RT boost, independent of risk category, were offered both alternatives of dose escalation. Based upon detailed information from the consultant oncologist and an oncology nurse, 50% of the patients made their own choice of the boost technique.

### 2.3. Statistical Analysis

Estimates of the probability of PSA relapse, occurrence of metastases, death, and persistent toxicities were calculated using the Kaplan-Meier method. Differences between risk groups were tested with univariate analysis using the log-rank test. The analysis of toxicities included only events occurring or persisting at least six months after the start of radiotherapy with the proton boost. In the analyses of treatment outcome, the followup was defined from the date patients started radiotherapy. All endpoints were studied separately and patients were censored at the time of occurrence of a competing event that prohibited the occurrence of the event of interest. Multivariate analysis was performed using Cox proportional hazards model and results are presented as hazard ratios with 95 percent confidence intervals. A *P* value ≤ 0.05 was considered as statistically significant. Actuarial estimates of the prevalence of persistent toxicities were calculated using the method proposed by Pepe et al. [33].

## 3. Results

**Figure 1:**
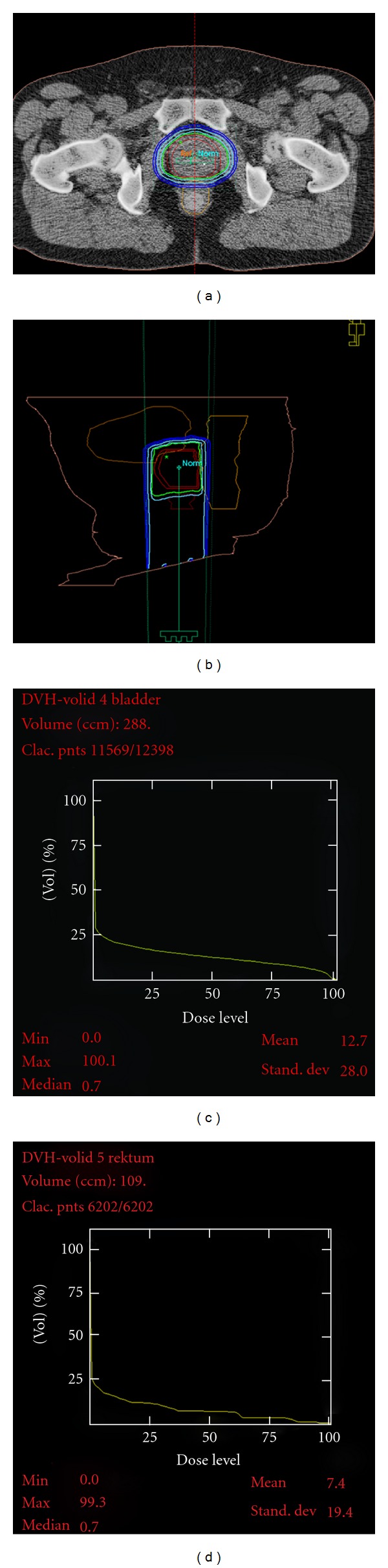
The planned dose distribution (panel a, b) for a patient receiving proton boost when using the rectal retraction rod and with dose volume histograms for bladder and rectum (panels c, d). The isodose levels shown are 95%, 90%, 70%, 50%, and 30%. The outer wall of the rectum is drawn by a yellow line.

A typical dose distribution with proton boost where the rectal retraction rod was used is shown in Figure 1(a).  Figure 1(b) shows a sagittal section of the dose distribution, while Figures 1(c) and 1(d) illustrate dose volume histogram (DVH) for the bladder and rectum, respectively.

### 3.1. Overall Survival and Disease-Specific Survival

**Figure 2:**
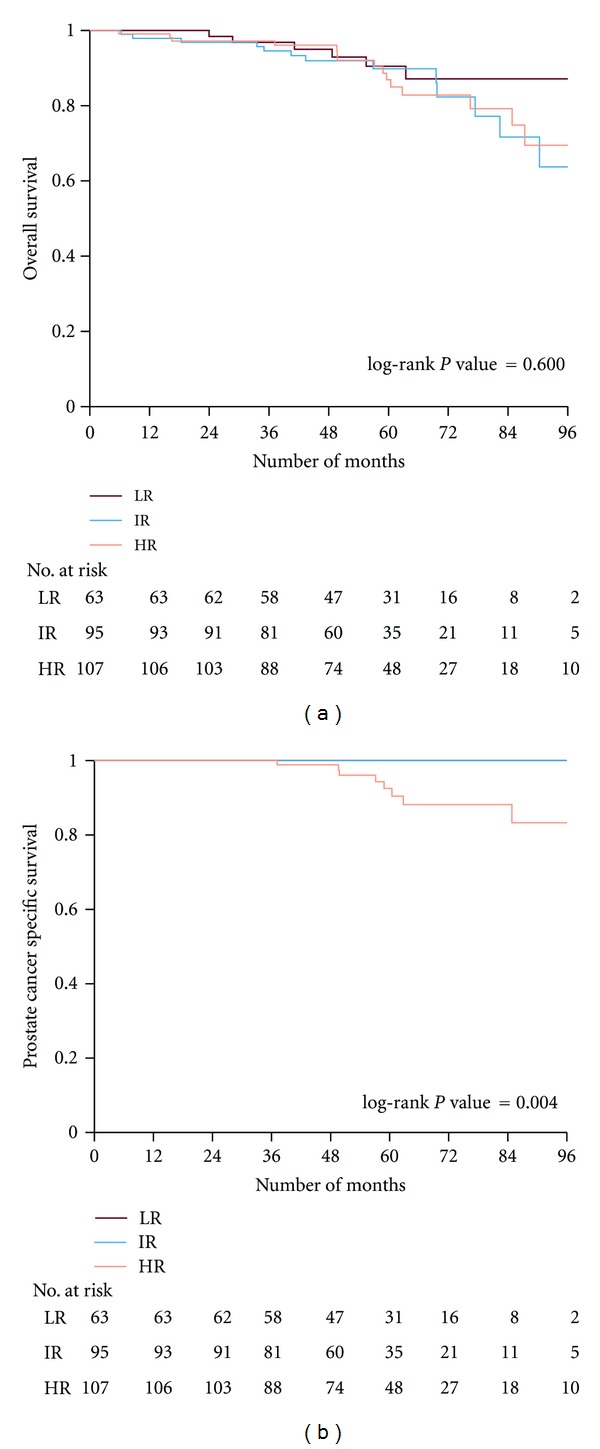
Kaplan-Meier curves illustrating the overall (panel a) and the prostate cancer-specific survival (panel b) of patients according to risk groups. The number of patients at risk is shown at different time intervals. LR: low risk, IR: intermediate risk, HR: high Risk.

The overall and prostate cancer-specific survival, by risk category and the number of patients at risk at different time intervals were estimated by Kaplan-Meier curves (Figures 2(a) and 2(b)). When the last assessment of the study parameters was performed the18th January 2012, 230 patients were still alive. The median follow-up time of the 265 patients was 57 months (range 5.8–109 months).

The 5- and 8-year overall survival of the whole patient cohort was 89% and 71%, respectively. The figures for low- intermediate- and high-risk groups at 5 years were 90%, 90%, and 87%, respectively, (*P* = 0.919). The overall survival at 8 years was 87%, 64%, and 69%, (*P* = 0.60), respectively. Only 8 patients, all being at high-risk, died due to PC after a median time of 58 months (range 35–83 months). The 5- and 8-year prostate cancer-specific mortality was 0% for the low and intermediate risk groups compared to 7% and 17% for the high-risk group, respectively, (*P* = 0.004).

### 3.2. PSA-Relapse and Distant Metastases

**Figure 3:**
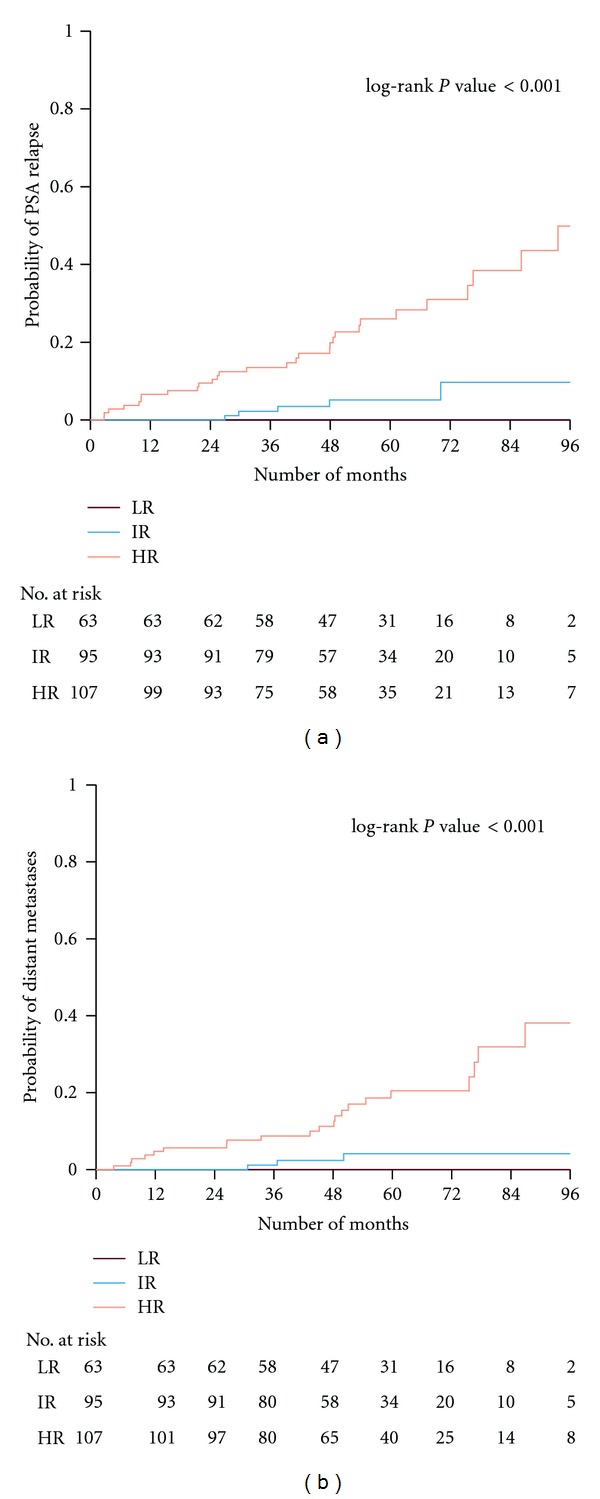
The probability of PSA-relapse (panel a) and developing distant metastases (panel b) according to risk groups is presented. The number of patients at risk is shown at different time intervals. LR: low risk, IR: intermediate risk, HR: high risk.

The probability of PSA-relapse according to the risk category is presented in Figure 3(a). The 5-year actuarial PSA relapse was 0%, 5%, and 26%, for low-, intermediate- and high-risk groups, respectively. At 8 years the corresponding figures were 0%, 10%, and 50%, respectively, (*P* < 0.001). Univariate analysis has demonstrated that tumor classifications of T3 (*P* < 0.001), T4 (*P* < 0.001), Gleason score (*P* < 0.001), and PSA at diagnosis (*P* < 0.001) were strongly significant in predicting PSA-relapse. Multivariate analysis revealed that clinical stage T3 (*P* = 0.003), T4 (*P* = 0.012), and Gleason score (*P* = 0.038) were significant predictors of PSA relapse (Table 2). The rectal retraction rod intended to reach improved target coverage did not demonstrate a significant impact on the risk of PSA recurrence.

So far, distant metastases (abdominal lymph nodes and bone metastases) have been diagnosed in 24 patients (9%) at a median time of 44.2 months (range, 3.5–86.9 months) during the followup. Distant and local metastases were evaluated upon a PSA relapse by radiological imaging, such as conventional examinations with bone scintigraphy, MR of the skeleton, and thoracic-abdominal CT. Two patients had local recurrence (one from the intermediate and one from the high-risk group) that was confirmed with histopathology.  Figure 3(b) shows the probability to develop distant metastases according to the risk category. The 5-year probability of distant metastases was 0%, 4%, and 20%, for the low-, intermediate-, and high-risk patients. At 8 years the figures were 0%, 4%, and 40%. Univariate analyses showed that T3 (*P* < 0.001), T4 (*P* = 0.015), Gleason score (*P* < 0.001), and pretreatment PSA (*P* = 0.024) were predictive factors for the development of distant metastases. However, the multivariate analysis could only confirm a predictive value for the T3 stage (*P* = 0.01) and the Gleason score (*P* = 0.037), (Table 3).

### 3.3. Persistent GU and GI Toxicities

At the latest followup, grades 2, 3, and 4 GU-symptoms according to the RTOG scale were scored in 29 (11%), 19 (7%), and 6 (2%) of the patients, respectively. Cox proportional hazard model showed that grade 1 (*P* = 0.001) or grade 2 (*P* < 0.001) GU symptoms at baseline, before start of neoadjuvant ADT or RT, compared to symptom-free patients was a strong predictive factor for developing GU-toxicity. Based upon these findings we decided to separate the toxicity analyzes according to pre-treatment symptoms: patients with grade 0 versus patients with grade 1 symptoms, that is, no medication meaning RTOG grade 1.

**Figure 4:**
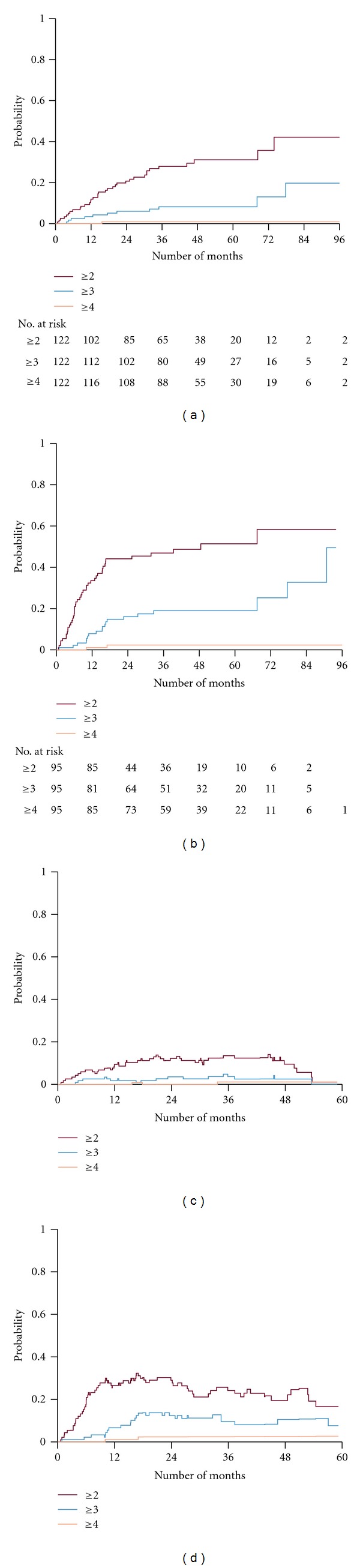
Cumulative probability of a genitourinary (GU) event of given grade, when base line GU level is grade = 0 (panel a) and baseline level GU level is grade = 1 (panel b). (Panels c and d) illustrate the actuarial prevalence of GU events of a given grade when baseline GU is grade = 0 and grade = 1, respectively. Numbers of patients at risk are shown at different time intervals.

The estimation of cumulative probability of toxicity was increasing over the 5-years followup. Concerning the baseline symptom-free patients (grade 0 in 122 patients) the actuarial development of grade ≥ 2, grade ≥ 3, and grade 4 toxicities is shown in Figure 4(a). The cumulative probability of GU toxicity grade ≥ 2 was analyzed according to the RTOG classification. The probability was for each grade 31%, 8%, and 1%, respectively, at 5 years. No significant predictive factors for toxicity could be identified in the uni- and multivariate analysis of GU symptoms-free patients at baseline (Table 4).

Concerning the patient cohort with mild untreated symptoms at baseline (grade 1 in 95 patients) the actuarial development of grade ≥ 2, grade ≥ 3, and grade 4 toxicities is shown in Figure 4(b). The probability for each grade was 51%, 19%, and 2%, respectively, at 5 years. The cohort with mild symptoms at baseline (grade 1) exhibited a significantly higher degree of toxicity compared to the cohort without symptoms before treatment (*P* = 0.001). Multivariate analysis of the group with grade 1 pre-treatment symptoms showed that TUR-P before radiotherapy (*P* = 0.018) and the PTV volume for the proton boost (*P* = 0.021) were significant predictive factors in developing GU side effects (Table 5). However, smoking and diabetes were not. Furthermore, the prostate cancer risk category did not either demonstrate a significant impact on GU toxicity.

In order to consider the fact that symptoms in some patients resolve completely, either spontaneously or after medical or surgical intervention, we did an estimation of the actuarial prevalence using Pepe's formula [33]. Again, this analysis was performed separately for patients with grade 0 versus grade 1 symptoms at baseline, before any treatment intervention of the prostate cancer (Figures 4(c) and 4(d)). In this prevalence analysis we were able to identify a decline in symptoms over a followup of 5 years. For baseline symptom-free patients the prevalence at 3 years was 13%, 4%, and 1% for grade ≥ 2, grade ≥ 3, and grade 4, respectively. At 5 years this group had a prevalence of 1%, 1%, and 1% for grade ≥ 2, grade ≥ 3, and grade 4, respectively. For patients with mild untreated symptoms at baseline, grade 1, the prevalence at 3 years was 24%, 10%, and 3% for grade ≥ 2, grade ≥ 3, and grade 4, respectively. At 5 years this group had a prevalence of 17%, 8%, and 3% for grade ≥ 2, grade ≥ 3, and grade 4, respectively.

Those 6 patients developing grade 4 GU toxicities had a significantly larger median prostate volume of 55 cc (range: 45–107 cc) compared to those with no grade 4 toxicities, having a median volume of 35 cc (range: 14–120 cc) (*P* = 0.05).

**Figure 5:**
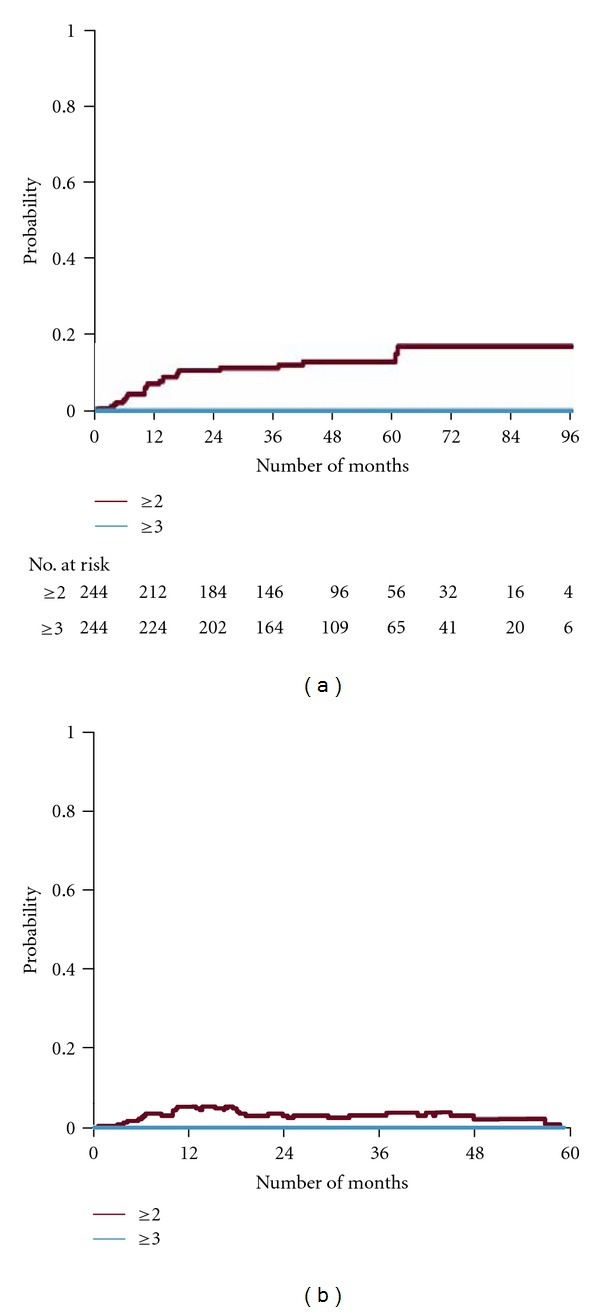
Cumulative probability of a gastrointestinal (GI) event of a given grade in patients with a baseline GI grade = 0 (panel a). Numbers of patients at risk are shown at different time intervals. (Panel b) illustrates the actuarial prevalence of a given GI grade in the same group.

Concerning GI-toxicity, the majority of patients had no symptoms at baseline (grade 0 in 244 patients). Only 15 patients had grade ≥ 1 and 5 patients had grade ≥ 2 symptoms. Neither grade 3 nor grades 4 GI toxicities were registered at baseline. For the baseline symptom-free patients the actuarial cumulative probability of GI toxicity grade ≥ 2 and grade ≥ 3 at 5 years was 10% and 0%, respectively, (Figure 5(a)). Multivariate analyses based upon the cumulative incidence demonstrated that diabetes was a significant predictive factor for the development of GI-symptoms grade ≥ 2, (*P* = 0.016) (Table 6). Furthermore, neither the proton PTV boost volume nor the use of the rectal retraction rod or smoking was identified as predictive factors for GI-toxicity.

The actuarial prevalence of GI symptoms for the symptom-free patients at baseline (grade 0 in 244 patients) is shown in Figure 5(b). The prevalence at 3 years was 3% for grade ≥ 2 and 0% for grade ≥ 3. The corresponding figure at 5 years was 0% for grade ≥ 2.

### 3.4. Neoadjuvant and Rogen Deprivation Therapy

Separate bivariate analysis was performed on the effect of neoadjuvant androgen deprivation therapy per risk category for all events of interest, that is, PSA relapse, distant metastases, overall survival, cancer-specific survival, and GU and GI toxicities. N-ADT had no significant effect on any of the events investigated (all *P* values were larger than 0.8).

## 4. Discussion

Here we present the outcome of a newly designed hypofractionated regimen delivered with proton boost as an alternative to HDR-BT boost, in order to achieve dose escalation in the use of radiotherapy for cure of prostate cancer. The results of this study demonstrate that treatment with hypofractionated proton boost combined with EBRT to a dose level of 87 Gy is well tolerated in patients with localized prostate cancer. The 5- and 8-year prostate cancer-specific mortality was 0% for the low and intermediate groups compared to 7% and 17% for the high-risk group, respectively, (*P* = 0.004). The 5-year actuarial PSA relapse was 0%, 5%, and 26%, for low-, intermediate- and high-risk groups, respectively. At 8 years the corresponding figures were 0%, 10%, and 50%, respectively (*P* < 0.001).

Two randomized studies have been performed investigating the outcome of dose escalation with a proton boost. In the first study published by Shipley et al. [3] using a perineal beam, with a rectal probe, comparing 75.6 GyE versus 67.2 GyE. Only the subgroup with poorly differentiated tumors experienced significant benefit of the higher dose. The GU-toxicity was similar for both doses, ending up with a persistent rate of urethra strictures less than 5%. The dose dependent cumulative incidence of rectal bleeding grade 2 was 32% from 5 years and onwards after 75.6 GyE, but the persistent rate was reduced to 9%.

The second study ACR-9509 run by two centres used different boost technique. At Loma Linda University Medical Centre, patients were treated in supine position with opposed lateral 250 MeV proton beams. At Massachusetts General Hospital, patients were treated in lithotomy position with a single perineal 160 MeV proton beam. The 5-year biochemical progression-free survival was 95% and 80% for the low-risk and intermediate-risk groups, respectively, after 79.2 GyE in 1.8 GyE per fraction, superior to the lower dose level [9]. The actuarial incidence of GU-toxicity was close to 20% at 5 years, independent of dose. The GI-toxicity increased with dose escalation. The 5-year cumulative incidence grade 2 was 17%.

In the report of the long-term outcome of IMRT dosage to 81 Gy, the 5-year PSA progression-free survival was higher than 95%, 90% and 70% for low-, intermediate-, and high, risk categories, respectively. The 5- year cumulative incidence of grade 2 GU-toxicity and GI-toxicity was 10% and 4%, respectively [16].

The previously reported outcome for the HDR-BT boost by Åström et al. [34] for PSA relapse at 5 years was 8%, 12%, and 39% for low-, intermediate-, and high-risk patients, respectively. One reason for the slightly better outcome with the proton boost in comparison with HDR-BT boost might be the wider margin around the prostate applied with the protons. In our study MRI was used to help the delineation of the prostate. However, fusion of the MRI and dose planning CT scans were not possible due to the different patient positioning of the two investigations for patients receiving proton boost. The more generous margin with the proton boost due to CT planning might imply a satisfactory coverage of the CTV even without the rectum retraction rod in place. That is probably one of the reasons why we do not see any significant impact of the rod on the PSA relapse so far. More events of local failure have to be awaited for final conclusion on this issue.

The outcome in the PSA progression-free survival for high-risk patients in our study could still be improved by adding adjuvant IMRT to the pelvic nodes as well as adjuvant androgen deprivation therapy (ADT) up to 3 years. The issue of elective pelvic lymph node irradiation is not definitively solved. Its impact is at best modest, but should be considered for patient with a high risk of regional disease [35]. The use of ADT for several years is not either clear cut, however, it might be indicated for high-risk patients [36]. Since the beginning of 2008 only high-risk patients were prescribed N-ADT and recommended adjuvant ADT for 3 years in our patient group. New evidence does support this strategy [37, 38].

Concerning the side effects scored according to the RTOG scale, we evaluated both the cumulative incidence and the prevalence. As expected the cumulative incidence increased over the followup both for GU and GI toxicities. However, in contrast to the incidence, the prevalence declined significantly with followup and reached noticeable low numbers for any grade of GU-toxicity. The estimated prevalence of GU-toxicities in symptom-free patients at baseline were 1% for both grade 2, grade 3, and grade 4 at 5 years. The corresponding figures for patients with mild GU symptoms at baseline were 17%, 8%, and 3% for grade 2, grade 3, and grade 4 at 5 years, respectively. The estimated prevalence for any grade (more than grade 1) of GI-toxicity at 5-years was 0%. More toxicity reports concerning radiotherapy of prostate cancer have also revealed a decline of symptoms both from the genitourinary and gastrointestinal tract, beginning a couple of years following RT. These observations were recently reviewed by Budäus et al. [39].

Our study group receiving proton boost has included prostate cancer patients with volumes over 100 cc, T4 tumors and patients with grade 2 and grade 3 GU-symptoms before treatment start. Similarly to our findings it was previously shown by others that patients with a large prostate volume (>50 cc) had a higher rate of acute grade 3 GU-toxicity (*P* = 0.002) [40]. Mild pretreatment symptoms from the genitourinary tract (RTOG grade 1) were a strong predictive factor for persistent GU-toxicity. Moreover, for this subgroup of patients TUR-P and the PTV volume for the proton boost were significant predictive factors.

The GI-toxicity in our study group was substantially lower than that usually reported [41]. The prevalence of GI-toxicity was also declining with prolongation of followup from 3 to 5 years, indicating a slow recovery for the rectal epithelium, and successively less probability of rectal bleeding. The maximum physical dose, generally located just behind the prostate gland is 70 Gy (Figure 1), but in terms of EDQ2 using *α*/*β* = 3 Gy it is 87 Gy. This is a higher limit than usually accepted [16, 17, 41]. Concerning the usefulness of straightening and retraction of the rectum by application of the rod, we could not notify any significant reduction in GI-toxicity in spite of a significantly lower median absorbed dose when the rod was used (*P* < 0.0001), data not shown. Lack of discrimination in GIside effects with and without the rectal rod is probably related to the few toxic events reported in our study group. However, diabetes was found to be a significant predictive factor for the risk of developing GI-toxicity (*P* = 0.016).

Remarkably, true late GI- and GU-toxicities were not observed in our patient cohort with a median follow-up of almost 5 years. True late radiation damage inflicted to cell populations in submucosal tissues is progressive over time, and the progression rate is strictly dose dependent [42, 43]. The lower the dose the longer the latency is to reach a tissue damage that will give a defined symptom. One example is urethra strictures, which might appear very late as a consequence of tissue fibrosis beneath the uroepithelium. The declining course of the prevalence in GU-toxicity between 3- and 5- year followup, must be interpreted as resolution of radiation damage to the uroepithelium, that is, a consequence of acute damage induced by RT, but with a delayed response in cell death and depletion of the uroepithelium. However, at a certain degree of cell loss in the epithelium an accelerated compensatory proliferation is triggered resulting at best in complete healing of the uroepithelial damage. Up to 5 years we have no signs of true late progressive damage in the genitourinary and gastrointestinal tract. The manifestation in our study and other reports, most clearly by Shipley et al. [3] that the prevalence is declining over 3 to 5 years for both GU- and GI-toxicity indicates a slow resolution of epithelial damage. Therefore, we underline that at least another 5-year followup is necessary for accurate conclusions of the true late progressive toxicity of this treatment regimen. Based on the reasoning above we propose that GU and GI symptoms during the first 5 years after the completion of radiotherapy should be described to epithelial damage rather than submucosal injury. The fractionation sensitivity for these tissues seems to be low; that is, the *α*/*β* is closer to 10 Gy rather than 3 Gy. The very low GU- and GI-toxicities in our study group could only be understood if the delivered physical dose of 70 Gy corresponds to an EDQ2 of 78 Gy with an *α*/*β* of 10 Gy instead of EDQ2 87 Gy with an *α*/*β* of 3 Gy.

## 5. Conclusion

The current data indicate that hypofractionated proton boost combined with EBRT is associated with an excellent clinical outcome and low rates of treatment toxicities. Bladder toxicities rather than rectal toxicities seem to be dose limiting and determined by epithelial damage over a follow-up time of 5 years. Long-term followup is necessary to evaluate the evolvement of any true late progressive and irreversible injury.

## Figures and Tables

**Table 1:** Patient characteristics.

(*N* = 265)	No. (%)		
Age at diagnosis			
Median (range)	65 (46–77)		
T-stage			
T1b	6 (2.3)		
T1c	89 (33.6)		
T2a	38 (14.3)		
T2b	22 (8.3)		
T2c	29 (10.9)		
T3a	64 (24.2)		
T3b	14 (5.3)		
T4	3 (1.1)		
Gleason score			
≤6	144 (54.3)		
7	86 (32.5)		
≥8	35 (13.2)		
PSA ng/mL			
Median (range)	10.0 (1.7–158.0)		
<10.0	127 (47.9)		
10.0–19.9	86 (32.5)		
≥20.0	52 (19.6)		

NCCN risk group		Median PSA (ng/mL) (range)	Median prostate volume (cc) (range)

Low risk	63 (23.8)	6.1 (3.0–9.6)	31.0 (19.0–90.0)
Intermediate risk	95 (35.8)	11.0 (1.7–20.0)	40.0 (14.0–113.0)
High risk	107 (40.4)	18.0 (3.6–158.0)	37.5 (14.0–120.0)
Smoker			
Yes	45 (17.0)		
No	212 (80.0)		
Unknown	8 (3.0)		
Diabetic			
Yes	33 (12.5)		
No	230 (86.8)		
Unknown	2 (0.8)		
TUR-P			
Yes	12 (4.5)		
No	251 (94.7)		
Unknown	2 (0.8)		
Rectal rod			
Yes	147 (55.5)		
No	118 (44.5)		
GU baseline			
0	122 (46.0)		
1	95 (35.8)		
2	38 (14.3)		
3	9 (3.4)		
4	0 (0.0)		
Unknown	1 (0.4)		
GI baseline			
0	244 (92.1)		
1	15 (5.7)		
2	5 (1.9)		
3	0 (0.0)		
4	0 (0.0)		
Unknown	1 (0.4)		

**Table 2:** Cox regression model—PSA relapse.

	Univariate analysis			Multivariate analysis		
	HR	(95% CI)	*P*	HR	(95% CI)	*P*
Age at diagnosis, continuous	0.99	(0.93–1.06)	0.741	0.99	(0.93–1.05)	0.726
T-stage						
T1	1.00	(ref.)	—	1.00	(ref.)	—
T2	4.25	(0.90–20.02)	0.067	3.63	(0.76–17.36)	0.106
T3	16.70	(3.92–71.11)	<0.001	9.67	(2.13–43.89)	0.003
T4	45.91	(6.41–329.02)	<0.001	14.80	(1.82–120.24)	0.012
Gleason score, continuous	2.05	(1.53–2.75)	<0.001	1.43	(1.02–2.00)	0.038
log-PSA, continuous	2.27	(1.54–3.34)	<0.001	1.40	(0.92–2.14)	0.115
Rectal rod						
No	1.00	(ref.)	—	1.00	(ref.)	—
Yes	1.99	(0.93–4.25)	0.074	1.21	(0.53–2.76)	0.653

**Table 3:** Cox regression model—distant metastases.

	Univariate analysis			Multivariate analysis		
	HR	(95% CI)	*P*	HR	(95% CI)	*P*
Age at diagnosis, continuous	1.00	(0.93–1.08)	0.966	0.99	(0.92–1.06)	0.713
T-stage						
T1	1.00	(ref.)	—	1.00	(ref.)	—
T2	2.08	(0.38–11.38)	0.397	1.94	(0.35–10.84)	0.451
T3	11.92	(2.75–51.62)	<0.001	7.79	(1.65–36.81)	0.010
T4	19.91	(1.79–221.62)	0.015	8.64	(0.67–111.96)	0.099
Gleason score, continuous	2.01	(1.44–2.80)	<0.001	1.48	(1.02–2.13)	0.037
log-PSA, continuous	1.76	(1.08–2.86)	0.024	1.08	(0.64–1.83)	0.776

**Table 4:** Cox regression model—GU grade 2 or higher^∗^.

	Univariate analysis			Multivariate analysis		
	HR	(95% CI)	*P*	HR	(95% CI)	*P*
Age at diagnosis, continuous	1.03	(0.96–1.11)	0.381	1.03	(0.96–1.12)	0.388
Risk group						
Low risk	1.00	(ref.)	—	1.00	(ref.)	—
Intermediate risk	2.07	(0.88–4.86)	0.094	1.90	(0.79–4.52)	0.149
High risk	1.05	(0.40–2.73)	0.921	0.91	(0.34–2.42)	0.850
Smoker						
No	1.00	(ref.)	—	1.00	(ref.)	—
Yes	0.94	(0.36–2.45)	0.900	1.04	(0.38–2.81)	0.943
Diabetic						
No	1.00	(ref.)	—	1.00	(ref.)	—
Yes	0.82	(0.25–2.69)	0.738	0.84	(0.24–2.90)	0.783
TUR-P						
No	1.00	(ref.)	—	1.00	(ref.)	—
Yes	2.40	(0.73–7.91)	0.152	2.00	(0.55–7.18)	0.290
log-PTV proton volume, continuous	2.49	(0.80–7.90)	0.116	2.88	(0.76–7.80)	0.135

^∗^Only including patients with GU grade 0 at baseline and not missing information on smoking, diabetes, TUR-P, and PTV volume (*N* = 117).

**Table 5:** Cox regression model—GU grade 2 or higher^∗^.

	Univariate analysis			Multivariate analysis		
	HR	(95% CI)	*P*	HR	(95% CI)	*P*
Age at diagnosis, continuous	0.97	(0.92–1.02)	0.249	0.97	(0.92–1.03)	0.276
Risk group						
Low risk	1.00	(ref.)	—	1.00	(ref.)	—
Intermediate risk	0.89	(0.41–1.97)	0.781	1.01	(0.45–2.27)	0.988
High risk	0.60	(0.26–1.38)	0.228	0.58	(0.25–1.36)	0.212
Smoker						
No	1.00	(ref.)	—	1.00	(ref.)	—
Yes	1.02	(0.51–2.02)	0.960	0.83	(0.40–1.71)	0.611
Diabetic						
No	1.00	(ref.)	—	1.00	(ref.)	—
Yes	1.54	(0.68–3.46)	0.297	1.89	(0.82–4.39)	0.136
TUR-P						
No	1.00	(ref.)	—	1.00	(ref.)	—
Yes	2.99	(0.91–9.81)	0.071	3.94	(1.12–13.88)	0.033
log-PTV proton volume, continuous	0.33	(0.13–0.84)	0.020	0.28	(0.10–0.76)	0.013

^∗^Only including patients with GU grade 1 at baseline and not missing information on smoking, diabetes, TUR-P, and PTV volume (*N* = 92).

**Table 6:** Cox regression model—GI grade 2 or higher^∗^.

	Univariate analysis			Multivariate analysis		
	HR	(95% CI)	*P*	HR	(95% CI)	*P*
Age at diagnosis, continuous	1.00	(0.92–1.09)	0.984	0.99	(0.90–1.08)	0.743
Risk group						
Low risk	1.00	(ref.)	—	1.00	(ref.)	—
Intermediate risk	1.12	(0.37–3.43)	0.842	1.17	(0.37–3.71)	0.791
High risk	1.22	(0.41–3.65)	0.720	1.27	(0.42–3.85)	0.673
Smoker						
No	1.00	(ref.)	—	1.00	(ref.)	—
Yes	0.73	(0.22–2.46)	0.610	0.57	(0.16–2.05)	0.388
Diabetic						
No	1.00	(ref.)	—	1.00	(ref.)	—
Yes	3.23	(1.26–8.33)	0.014	3.51	(1.35–9.13)	0.010
Rectal rod						
No	1.00	(ref.)	—	1.00	(ref.)	—
Yes	1.41	(0.58–3.43)	0.453	1.36	(0.56–3.33)	0.499
log-PTV proton volume, continuous	1.68	(0.46–6.17)	0.433	1.94	(0.48–7.89)	0.355

^∗^Only including patients with GI grade 0 at baseline and not missing information on smoking, diabetes, rectal rod, and PTV volume (*N* = 236).
